# Cooperation in the Time of COVID

**DOI:** 10.1177/17456916231178719

**Published:** 2023-06-29

**Authors:** Jade Butterworth, David Smerdon, Roy Baumeister, William von Hippel

**Affiliations:** 1 School of Psychology, University of Queensland; 2 School of Economics, University of Queensland; 3 Research With Impact, Queensland, Australia

**Keywords:** cooperation, evolutionary psychology, information sharing, intragroup processes, reciprocity, reputation, social cognition

## Abstract

Humans evolved to be hyper-cooperative, particularly when among people who are well known to them, when relationships involve reciprocal helping opportunities, and when the costs to the helper are substantially less than the benefits to the recipient. Because humans’ cooperative nature evolved over many millennia when they lived exclusively in small groups, factors that cause cooperation to break down tend to be those associated with life in large, impersonal, modern societies: when people are not identifiable, when interactions are one-off, when self-interest is not tied to the interests of others, and when people are concerned that others might free ride. From this perspective, it becomes clear that policies for managing pandemics will be most effective when they highlight superordinate goals and connect people or institutions to one another over multiple identifiable interactions. When forging such connections is not possible, policies should mimic critical components of ancestral conditions by providing reputational markers for cooperators and reducing the systemic damage caused by free riding. In this article, we review policies implemented during the pandemic, highlighting spontaneous community efforts that leveraged these aspects of people’s evolved psychology, and consider implications for future decision makers.

Humans evolved to cooperate. Humans stand in stark contrast to their nearest cousins—the chimpanzees—who prefer to forage alone (Bullinger et al., 2011), who disguise the direction of their attention from each other rather than broadcasting it as people do (via the whites of their eyes; Tomasello et al., 2007), and whose cooperation reliably breaks down whenever individuals have the opportunity to monopolize the fruits of their joint efforts (Melis et al., 2006). The cooperativeness humans evolved to survive and thrive on the savannah underlies the human success story (von Hippel, 2018), but nature has not written humans a blank check. Along with a cooperative nature, humans also evolved tendencies toward tribalism, parochialism, and violence, and these more troubling aspects of human nature are amplified when people are under threat (Pinker, 2003).

In the current article, we begin with a brief discussion of the circumstances that facilitated cooperation in ancestral environments and how cooperation breaks down when these conditions are absent. We then examine how COVID-19 policies either leveraged human cooperative tendencies or undermined them, bringing out the best and worst in human nature. The fault lines of human cooperation are visible under the best of circumstances, primarily because people can benefit by exploiting the cooperation of others without contributing themselves, but the risks to human society become particularly evident during episodes such as the COVID-19 pandemic. A better understanding of how humans evolved to cooperate could facilitate policymaking during the next pandemic.

## Three Ingredients for Successful Cooperation

There are three critical factors that promote cooperation in humans and other social animals. First, when individuals are known to each other, they gain important reputational benefits from being helpful. Cooperative people are preferred relationship partners in every society on earth, so much so that cooperativeness often trumps other important qualities, such as competence (Bird & Power, 2015). Even vampire bats show clear preferences in whom they help (by regurgitating blood to feed unsuccessful hunters) and from whom they are willing to receive help (Carter & Wilkinson, 2015). In contrast, when individuals are unknown to each other, there is much less incentive to help; experiments on deindividuation have provided some of the clearest examples of the costs of being anonymous (Diener et al., 1976).

Second, when relationships are relatively enduring, they create much more potential for reciprocal helping because recipients cannot always repay helpers in the immediate context in which help is needed. People who expect to see each other on a regular basis are less concerned about whether a favor will be repaid in the short term because opportunities for reciprocation almost always emerge eventually. Indeed, long-term and tight-knit communities benefit from both reciprocal altruism and indirect reciprocity because everyone helps each other, secure in the knowledge that the reputational benefits accumulate over time (Yoeli et al., 2013). Such circumstances stand in contrast to the one-shot interactions that are common in urban environments (and some forms of social media), where people know they can exploit each other with impunity.

Being identifiable and enmeshed in long-term relationships are critical ingredients for cooperation to emerge and be sustained, but they both depend on a third factor, which is that the costs to the helper must be less than the benefits to the recipient—that is, helping must be positive-sum (Ent et al., 2020). Humans and other animals are very attentive to the relative costs and benefits of cooperation and are most likely to offer help when the benefits outweigh the costs (Tooby & Cosmides, 1996). Fortunately, most instances of cooperation are indeed positive-sum because the law of diminishing marginal utility ensures that surplus goods are more valuable to people who have none than to people who have plenty.

Humans have an additional and incredibly potent source of positive-sum helping that is not available to other animals, enabled by humans’ extraordinary capacity for information sharing. Because information is so valuable for a highly cognitive species, such as humans, and because it is so easily shared through language, humans have unparalleled opportunities to help each other in mutually beneficial ways. Thus, information sharing is one of the clearest signs of human cooperation, whereas information hoarding is one of the clearest signs that self-interest has trumped the interests of the collective (Baumeister et al., 2018).

Ancestral societies typically contained all three of these ingredients that promoted cooperation: Everyone was known to everyone else in their group, relationships tended to be stable and long-term, and the most common modes of societally mandated helping (e.g., sharing of successful hunts or extra goods) relied on the decreasing marginal returns of surplus food or resources to ensure that helping was always positive-sum (Boehm, 2009). Human helping is even more positive-sum in modern nation-states than it was in the past because of the increased availability of surplus goods and ease of information transmission, but people are often unknown to each other, and relationships are often short-term. In short, despite the opportunities they present, cities full of strangers have created a fundamental threat to human cooperation that never existed in the lives of their ancestors.

Nonetheless, crises can knit societies together, cultivating human cooperation that would not otherwise exist (Zaki, 2020). Natural and human-made disasters often bind communities together in solidarity, eliciting mutual aid among survivors and voluntary assistance from unaffected people (Kaniasty & Norris, 1995; Páez et al., 2007; Solnit, 2010). Particularly when crises create superordinate goals, the resultant social cohesion can override intergroup conflict as previous adversaries become collaborators (Sherif, 1958). Not all crises are equivalent, however, because pathogen threats in particular lead to social avoidance and intergroup conflict (Cashdan, 2012; McGovern & Vanman, 2020), as was evident in the increased anti-Asian prejudice during COVID-19 (e.g., Lantz & Wenger, 2023). With these competing forces in mind, it is important to consider how policies created in response to the COVID-19 pandemic facilitate cooperation by promoting positive-sum, reciprocal, and identifiable helping opportunities.

## Information Sharing as Positive-Sum Helping

Information sharing is a highly sustainable form of cooperation because everyone benefits from reciprocal exchanges. For example, scientists who make their preprints available or collate and disseminate information discovered by other researchers benefit when other scientists do the same, thereby creating a self-sustaining ecosystem that supports all members of the community. Perhaps the key feature of this form of cooperation is that it is particularly robust to free riders because information sharing is so easy and hence so positive-sum that it can be sustained if only a small percentage of the community contributes. Given the centrality of information sharing in modern human helping, we explore three forms of information sharing in response to the COVID-19 pandemic.

### Information sharing among scientists

The rapid spread and alarming death rate of the novel coronavirus triggered an urgent need for information, causing laboratories with relevant expertise to refocus their energies on prevention and treatment (Waltman et al., 2021). In response to this situation, Wellcome (2020) issued a statement that was signed by 160 organizations worldwide committing to the swift, wide, and open sharing of COVID-19-related data and research findings. Because the complexity of the problem required knowledge from various disciplines, it was clear from the outset that rapid progress toward a vaccine required investigators to share their early findings with one another (Brainard, 2022).

**Fig. 1.**
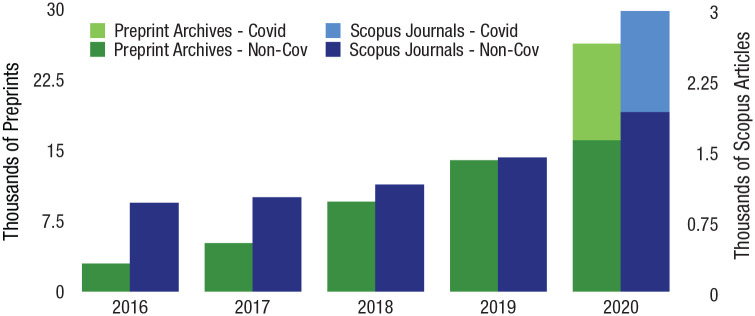
Growth in volume of articles shared on preprint archives (compared with the number of articles published in Scopus journals) between January and June of each year (adapted from Aviv-Reuven & Rosenfeld, 2021).

Although investigators often keep their early findings secret to give themselves a competitive advantage, the clear superordinate goals of developing evidence-based treatment and a vaccine led to increased information sharing in a variety of domains, such as the rapid increase in sharing of preprints when investigators began working on COVID-19 (see Fig. 1). Journal publishers responded to the crisis with similar urgency, launching the Covid-19 Rapid Review Collaboration Initiative (Open Access Scholarly Publishing Association, 2020), with the result that COVID-19 articles were reviewed and accepted by academic journals much faster than similar non-COVID-19 publications (Aviv-Reuven & Rosenfeld, 2021) and included more cooperative comments and less onerous requests made by peer reviewers (Horbach, 2021).^1^

**Fig. 2.**
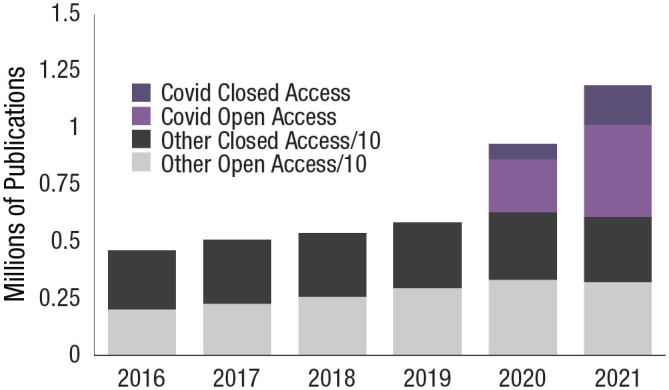
Open- versus closed-access publications (adapted from https://www.dimensions.ai/; Digital Science, 2018). Publication denoted as “other” are divided by a factor of 10 for ease of comparison.

Increased information sharing undoubtedly reflects self-promotion along with prosocial motives, but increases in the public availability of data (Larregue et al., 2020), collaboration (Duan & Xia, 2021; Lucas-Dominguez et al., 2021), open- versus closed-access publications (see Fig. 2), and even third-party sharing of other investigators’ findings (Peeples, 2022) all suggest increased cooperation on the part of the scientific community. For example, COVID-19 data dashboards and community pages were assembled to provide up-to-date findings, metrics, and reports to the public, researchers, and policymakers.^2^ Scientists also exploited the information-sharing capacities of social media platforms such as Twitter to synthesize other researchers’ findings into concise, collated posts that could be rapidly shared and understood by both experts and laypeople (Brainard, 2022).

**Fig. 3.**
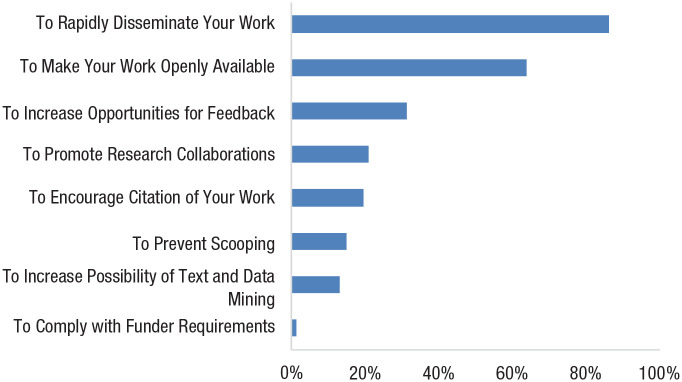
Reasons for posting COVID preprints (adapted from Waltman et al., 2021).

Although competition among scientists undoubtedly spurred many of these activities, the importance of the problem far outweighed the reputational benefits that laboratories might gain by hoarding information and solving the problem first. Thus, the global community of scientists began to collaborate as if they were all investigators in the same lab—with individuals assuredly hopeful that their contributions would be recognized by their peers but with these concerns secondary to the overarching goal that the team itself should succeed. For example, 86% of scientists reported that they had prepared COVID preprints for public archiving at least in part to enable the rapid dissemination of research (see Fig. 3). This sharing of preprints proved to be critical in the development of pandemic policy, with preprints more commonly referenced in World Health Organization (WHO) policy guidelines than published articles (Waltman et al., 2021).

Of course, not all aspects of interdisciplinary communication were cooperative. Researchers also competed for access to policymakers, with each discipline attempting to influence policy in a manner consistent with its priorities. Perhaps the most noteworthy example of such interdisciplinary competition was between economists—whose first concern is often pursuit of economic growth/avoidance of a recession—and epidemiologists—whose first concern is often reducing transmission. Because of these different priorities, many economists argued that lockdowns incurred too steep of a price while epidemiologists typically rebutted that lockdowns were necessary to preserve public health (Murray, 2020). Competition between disciplines for access to policymakers is not necessarily problematic, however, because politicians and bureaucrats who are responsible for both public health and the economy can assess the problem from various levels of analysis.

Unfortunately, in this particular case, pitting health outcomes against financial outcomes may have been experienced by policymakers as a taboo trade-off, forcing them to decide between sacred and secular values (Tetlock, 2003). As a result, rightly or wrongly, the concerns of epidemiologists often trumped those of economists as governments made lockdown decisions. Despite warnings that lockdowns could also lead to loss of life and not just loss of economic output and employment (Atlas et al., 2020), lockdowns were widely endorsed in an effort to “slow the spread” (Gottlieb et al., 2020). It remains to be seen whether the costs of lockdowns in quality-adjusted life years lost to substance use, domestic abuse, suicide, untreated heart attacks, diabetes, and dementia, among other factors, may have outweighed the benefits gained in slowing transmission (Miles et al., 2021).

### Information sharing between scientists and the community

Because the benefits of information sharing from scientists to the community are dependent on acceptance of the scientists’ findings by the community, information sharing in such circumstances is not so much a form of cooperation as it is a process of norm creation and reinforcement (Hoffman & Yoeli, 2022). The persuasive intent of this form of one-way transmission carries with it certain risks because accuracy and nuance can become secondary to the underlying persuasive goal. The most notable instantiation of this risk is when scientists intentionally (“disinformation”) or unintentionally (“misinformation”) spread inaccurate information (Lazer et al., 2018; Scales et al., 2021), undermining the principle of positive-sum helping (i.e., sharing resources is cooperative only if the resources are helpful). The spread of misinformation and disinformation is not uncommon during public-health emergencies (Das & Ahmed, 2022) and appears to have been used by scientists to influence behavior during the COVID-19 pandemic (Nazar & Pieters, 2021).

Perhaps the most notable example of mis/disinformation sharing during the pandemic is when the Centers for Disease Control and Prevention (CDC) and WHO initially discouraged the public use of face masks to prevent the spread of the virus, citing limited and mixed evidence of their efficacy (Murray, 2020; Servick, 2020). It was later revealed that this recommendation was born, at least in part, from a supply shortage and prioritization of frontline health workers (Howard, 2020) and the concern that people would engage in risk compensation, whereby mask wearers might engage in fewer preventive behaviors such as social distancing and hand sanitizing (Murray, 2020).

The risks inherent in sharing inaccurate information became apparent in this case when scientists and public-health authorities later advocated for the use of face masks in public settings, sowing distrust and confusion among members of the public. This situation was further complicated by the recommendation that people should wear masks even if they had been vaccinated, presumably because enforcing mask wearing by everyone is easier than enforcing mask wearing by the unvaccinated (Hoffman & Yoeli, 2022). Such a recommendation might serve the immediate interest of public health, but it also communicates the underlying message that the vaccine is ineffective in preventing transmission of COVID-19, which could have the unintended consequence of increasing vaccine skepticism (independent of whether that implication proves to be true). Ironically, a recent Cochrane report now suggests that face masks might not slow transmission after all (Jefferson et al., 2023), a point to which we return later.

Situations such as these can be difficult to avoid because persuasion goals involve different dynamics from cooperation goals. Nonetheless, communication between scientists and the public does not need to be a one-way street because “citizen scientists” play an increasingly important role in information gathering. Thus, one way to increase the mutual trust and cooperation between scientists and the public would be to promote the sort of two-way information sharing between scientists and the public that is experienced among scientists. For example, when scientists share their latest findings with the public, including inconsistencies and unknowns, the public tends to be more trusting of their honesty (Petersen et al., 2021).

Such open information sharing can also clarify to the public that scientists need their help to answer pressing questions. For example, scientists can create databases that allow members of the public to enter information about themselves (e.g., their mask-wearing habits, the extent of their socializing, whether they have contracted COVID) to help scientists address important questions.^3^ In such circumstances, both sides are incentivized to provide information that is timely and accurate but also nuanced. Scientists are motivated to tell the public what they know and what they do not know, with the latter information motivating members of the public to help scientists address the unknowns. In essence, such an approach would build off the enormous success that wikis have had in leveraging the knowledge and goodwill of members of the public, particularly if such databases could be maintained with similar levels of moderation, control, and support as are available on successful sites such as Wikipedia.

### Information sharing among the community

Perhaps the most salient form of positive-sum cooperation during the pandemic was information sharing within the community. Citizens flooded public forums and social media platforms with information about the origins of the virus, government objectives, home remedies, alternative treatments, and the risks of vaccinating (Banerjee & Meena, 2021; Scales et al., 2021; Volkmer, 2021; Yang et al., 2021). Perhaps unsurprisingly, much of that information was inaccurate because people share information at similar rates from high- and low-quality sources (Cinelli et al., 2020). Cooperation breaks down when information sharing is disrupted by mis/disinformation, but the risk of mis/disinformation is not as high as it might seem; information sharing does not translate directly to information believing because people are more likely to share than to believe misinformation (Pennycook et al., 2020). Among the many widespread falsities were claims that the virus could be transmitted through 5G Internet (Das & Ahmed, 2022), treated with hydroxychloroquine (Rudolph et al., 2021) or ivermectin (“Ivermectin May Help Covid-19 Patients, but Only Those With Worms,” 2021), or even that the virus was itself a hoax crafted by the government to inject the community with location-tracking microchips disguised as vaccines (Scales et al., 2021).

Social media has enabled information sharing among the public with unprecedented reach and speed, allowing both accurate and false information to be accessed by millions before it can be fact-checked and, where appropriate, debunked. People’s cooperative proclivity to share information combined with the availability, rapidity, and reach of digital platforms quickly transformed the pandemic into an “infodemic” (Banerjee & Meena, 2021) in which nearly everyone was exposed to a jumble of accurate and inaccurate information. Social media companies and their parent corporations responded to this situation by promoting authoritative content from the CDC, WHO, and other relevant health officials; providing links to PubMed and Google Scholar when people shared misleading information; removing misleading advertisements and posts (and sometimes user accounts) that contained (or consistently shared) misinformation; and reducing recommendations of borderline content (Banerjee & Meena, 2021; Shu & Shieber, 2020).^4^

Although efforts at censorship often backfire (e.g., removed ads sometimes attract more attention on a new site with labels such as “Censored: The government doesn’t want you to see this”; Jansen & Martin, 2003), the policy of attaching authoritative links to misinformation leverages people’s natural tendency to double-check dubious claims (Mercier, 2020; Pennycook & Rand, 2021). Because information accuracy became more important during the pandemic, people became more careful about their information sources and more likely to search for information from diverse and authoritative outlets (Volkmer, 2021). By providing easy access to accurate information, these social media policies reduced the reactance brought about by censorship (which they also engaged in to some degree) and more generally dampened the widely reviled “Big Brother” aspects of social media platforms by showing trust in the users’ judgment while also giving them control over their own information exposure.

In addition to sharing virus-related information, social media platforms aided other forms of information sharing during the pandemic. For example, YouTube (2020) launched Learn@Home to assist parents of schoolchildren with remote learning, and Instagram (2020) launched media sharing—a feature that enables joint viewing of user content via video chat. Some social media influencers (personalities with large followings) were also engaged to distribute accurate information and encourage public-policy compliance (Volkmer, 2021).

## Reciprocity

There is typically more incentive to cooperate within small communities rather than large communities because there is more opportunity for delayed or indirect reciprocity when people have repeated interactions with one another. Small communities provide opportunities for repeated interactions through networks of well-known people in which prosocial acts can be remembered and reciprocated, whereas large communities more often host one-off interactions with strangers. Because of this asymmetry in opportunities for delayed or indirect reciprocity, small towns have greater potential than large cities to benefit from community-wide cooperation during the pandemic.

Perhaps in response to this situation, residential areas of all sizes saw the rapid creation of subcommunities in the form of neighborhood-level mutual-aid groups.^5^ These mutual-aid groups sprang up around the globe as volunteers used online spreadsheets to list their contact details and the types of assistance they could provide to vulnerable members of their community (Samuel, 2020). Operating under banners such as “Solidarity, Not Charity” (Kaba & Spade, 2020), such mutual-aid societies were able to overcome the “banker’s paradox” (Tooby & Cosmides, 1996), or the threat to cooperation that emerges when people are not well known to each other and the need is great (and hence the cost of help is high and the chance of repayment is low). By focusing their efforts locally, these subsocieties allowed people to pool their resources and efforts, thereby reducing the burden on individual helpers while increasing the chances that people who were providing help to others would have their efforts recognized.

These spontaneously generated mutual-aid societies have numerous advantages that facilitate helping and cooperation. They allow people to choose the help they are most able to give and advertise their offers of help in a context that avoids the impression they are simply showing off, they leverage the enormous capabilities of the Internet to enable people to respond rapidly to the needs of others in a well-organized fashion that avoids effort duplication, and they create in-groups in which people feel a mutual connection and sense of belonging. Consistent with these advantages, mutual-aid societies and other forms of helping friends and neighbors played an important role in numerous communities (Sitrin & Sembrar, 2020). Although people often retreat to their closest in-groups and family members in the face of pathogen threats such as pandemics (Cashdan, 2012; Fincher & Thornhill, 2012), localized mutual-aid societies proved to be highly effective in countering that threat (at least in the case of the COVID-19 pandemic, in which death rates from the illness were not astronomical).

Anonymity stymies reciprocity, so it is no surprise that anonymity can bring out the worst in people when faced with major threats such as pandemics. A common consequence of anonymity is the “tragedy of the commons,” whereby people overconsume resources because they worry that others will take advantage of the situation and they will be exploited if they show restraint out of concern for the public good (Milinski et al., 2002). In the case of the pandemic, the obvious example of such behavior was supermarket hoarding, with shelves across America and in many other countries suddenly emptied of staple goods that were purchased in excess of what was needed at the moment.

Supermarkets and other organizations adopted various strategies to offset this problem, some of which were facilitated by government COVID policies that allowed collaborative practices among businesses that might otherwise be perceived as collusion (e.g., allocating customers or geographic areas to different businesses, serving some subsets of customers, such as elderly people, at specially designated times; Howarth & Alexander, 2020). Such policies increase the risk of a variety of anticompetitive behaviors, but they have the clear advantage of allowing corporations to cooperate with each other in the same manner that individuals do, in service of the immediate and emergency needs of their customers.

One of the greatest threats to cooperation is free riding, which occurs when a subset of the populace benefits from the helpful behaviors of others without contributing themselves. If free riding becomes sufficiently widespread, people stop contributing to the public good for fear of being exploited. This problem is avoided in circumstances in which reciprocity is direct and immediate but not when behaviors or individuals are difficult to identify, when reciprocity is delayed, when behavior helps others in general rather than specific others, and so on. A variety of policies were implemented during the pandemic to reduce free riding. Because one of the most notable instantiations of free riding during the pandemic is relying on herd immunity rather than getting vaccinated oneself, many such policies were aimed at vaccination. In Australia, for example, an 85% vaccination rate was achieved (compared with 69% in the United States) in part by limiting access to public venues, bars, and other sources of entertainment to people who were not vaccinated. Such policies are not equally palatable in different countries, but people around the world enacted their own personal forms of third-party punishment when they encountered free riders, for example by unfriending them on social media, refusing to socialize with them, or notifying authorities about parties that broke lockdown rules.^6^

## Reputation

Because humans evolved to cooperate, cooperation itself can be intrinsically rewarding (Tauer & Harackiewicz, 2004). Nonetheless, humans did not evolve to cooperate with everyone, nor did humans evolve to cooperate all the time, and thus policies that leverage aspects of humans’ evolved psychology to enhance cooperation can be very useful when cooperation is required across boundaries that might normally attenuate it (e.g., cooperating with out-groups or anonymous others). Under such circumstances, reputational markers can provide an important incentive to cooperate when people would otherwise be disinclined to do so (Hoffman & Yoeli, 2022).

Many responses to the pandemic involve public-facing behaviors and thus have the advantage that simply engaging in them provides people with the necessary reputational markers (Shumsky et al., 2021). For example, socially distancing in supermarket lines by standing on labeled stickers,^7^ scanning a QR code to verify one’s vaccination status before entering a public venue, and wearing masks are visible to others in the vicinity, leading many people to engage in these behaviors who might otherwise be disinclined. But many important forms of cooperation during a pandemic do not have an obvious behavioral signature—with vaccination being the most notable example—allowing people to forgo these important forms of cooperation without suffering reputational costs. In circumstances such as these, policies that make visible private behaviors have the best chance of inducing cooperation (Yoeli et al., 2013). For example, many vaccination sites offered complimentary stickers as proof of vaccination, and social media platforms launched digital stickers to the same effect (Hutchinson, 2021). Many people spontaneously advertised these sorts of behaviors on their social media sites via selfies at vaccination sites, for example. These public instantiations of private behaviors serve as signals of one’s cooperativeness, with such signals playing an important role in the development and maintenance of cooperation among humans and many other animals (Számadó et al., 2021). In the context of the COVID-19 pandemic, such behaviors have the potential to create virtuous cycles of cooperation by strengthening norms in support of public health.

Note, however, that such reputational strategies are most effective among people who are at least indifferent about norms of cooperation. People who are actively hostile to them, such as anti-vaxxers, care much more about the opinions of their like-minded group members than they do about the opinions of the general public. As a consequence, making visible forms of pandemic cooperation, such as vaccination, is highly unlikely to cause people who are hostile to vaccination to change their mind and might even make them less likely to get vaccinated because they are primarily concerned about signaling their identity and group membership to other anti-vaxxers. In such circumstances, increasing the visibility of cooperation with public-health mandates such as vaccines is likely to reduce cooperation only among actively hostile subcommunities.

Indeed, reputational markers of noncooperation are just as important to the subgroup of people who are against lockdowns, vaccine and mask mandates, and so on as they are to people who believe that these public-health behaviors are an important shared responsibility (Funkhouser, 2022). One need only consider the many public protests over vaccination and mask mandates (Lange & Monscheuer, 2022) or the media personalities who openly spread misinformation and disinformation about vaccinations or treatment options, sometimes even to the point of their own hospitalization and death from the virus (“Deaths of Anti-Vaccine Advocates From COVID-19,” 2023), to realize not only that reputations are of the utmost importance but also that reputational strategies are directed primarily toward others who are regarded as in-group members (Winterich et al., 2013).

The public-health challenges that are created when reputational markers are directed at different subcommunities is a costly risk, but it is important to keep in mind that a plurality of approaches can be valuable when the science itself is not settled. In the case of COVID-19, the world was faced with a novel virus that spread with incredible rapidity, with the result that there was no clearly correct approach that everyone should follow. Vaccination is almost always a public good, but new vaccines may or may not be effective or safe over the long term. Protecting others by wearing a mask seems like a low-risk cooperative approach, but masks may or may not be effective in slowing the spread of disease.

If all humans held the same values and adopted the same approaches to the COVID-19 pandemic, there would be very little data on the efficacy of the various approaches. But because there was enormous disagreement about the best approach, with resulting variability in the adoption of different strategies, it seems likely that there will soon be a mountain of evidence about which practices were and were not efficacious in dealing with the virus and in creating public cooperation in limiting its spread. The Cochrane report (Jefferson et al., 2023) suggesting that masks might not have reduced the spread of COVID-19 is a clear example of such data, although even in this case, much more evidence is needed (and will likely soon be available). From this perspective, the misinformation and disinformation of various individuals and special-interest groups might well have served the long-term interests of science even though it created confusion and was often disruptive of the cooperative efforts of various communities.

## Policy Implications and Conclusions

In this brief review, we explored how to leverage understanding of the nature of cooperation to facilitate public health during crises such as pandemics. In service of that goal, we conclude by highlighting three areas in which policies enhanced (or could have enhanced) cooperation by addressing key issues raised in this review, following which we highlight important questions for future research.

### Positive-sum helping

Cooperation is positive-sum when the benefits to the receiver are greater than the costs to the giver. Policies can enhance cooperation by amplifying these properties of positive-sum helping: by making help easy to give, by emphasizing its benefits, and by ensuring that it is indeed helpful. Information sharing was the most widespread example of positive-sum helping at every level of the community during the pandemic.

### Information sharing among scientists

The pandemic itself created superordinate goals that enhanced information sharing, but a number of policies facilitated this process, such as the Wellcome statement on data sharing, editorial practices of rapid review, and the creation of institutional data dashboards. Future policies could enhance interdisciplinary collaboration and information sharing among scientists through the early development of official data dashboards and virtual research teams, allowing diverse fields to collaborate and contribute to public-health decisions.

### Information sharing between scientists and community

Information sharing is more cooperative when it flows in both directions. Policies that encourage scientists and the community to share accurate information with each other to their mutual benefit can minimize the temptation to share inaccurate information in service of persuasive goals. Future policies could create wikis for the purpose of data sharing between scientists and the public with the goal of allowing scientists to share discoveries as well as questions that encourage the community to provide accurate information.

### Information sharing among the community

Social media companies enhanced the quality of information sharing by connecting mis/disinformation that was shared among the community with fact-based sources that people could access themselves. This strategy appears to be more effective than censorship or de-platforming of purveyors of mis/disinformation because it leverages the widespread tendency to search for objective information when confronted with important decisions. Future policies could apply these successful strategies used by social media platforms to a variety of information outlets, with the goal of making authoritative information available on sites that contain extensive misinformation or information that is not yet fact-checked.

### Reciprocity

The creation of mutual-aid groups via Google sheets and other methods fostered the emergence of small, reciprocally cooperative communities that provided their members with a great deal of assistance. Future policies could benefit from the creation of mutual-aid apps (similar to those used in the gig economy) that connect members of the community in reciprocally beneficial, timely, and local exchanges.

### Reputation

Enhancing the visibility of cooperation reduces free riding but also leads to counter-normative behavior among dissenting subgroups. It is unclear how future policies could reduce free riding by enhancing visibility of cooperative behaviors such as vaccination without immediately creating dissenting subcommunities.

### Future research

Although these implications follow directly from what is known about cooperation and the COVID-19 pandemic itself, because of the recency of the pandemic, it is not yet clear which strategies were most effective in reducing transmission, nor is it clear which strategies were most useful in leveraging cooperation. Addressing these issues will be a critical area of future research because the potential value of public-health strategies will emerge only if people are willing to cooperate with one another in service of public-health goals. Thus, answers to questions such as the following are needed: Did the scope of scientific sharing accelerate discovery, did it enhance the trust people have in science, and did it promote the dissemination of new information to the public? Did reputational strategies enhance vaccination rates, social distancing, mask wearing, or any other health behaviors that were advocated by public officials?

Despite these important unanswered questions, the data that are currently available suggest that grassroots efforts by local communities were often the most effective in leveraging aspects of humans’ evolved cooperative tendencies to enhance the public good. Although the government and major policy bodies often have more power than spontaneously assembled groups of individuals, the former can be much more difficult to mobilize. In the case of official government policies in the United States, not only was there a lack of coherence across the different states, but there are now a number of lawsuits and legislative efforts aimed at reducing the power of local authorities to enforce public-health decisions, such as mask mandates or school closures (Weber & Achenbach, 2023). These responses to the pandemic emphasize that there are enormous barriers in pluralistic societies to any official responses to public-health crises—again emphasizing the utility of grassroots approaches. Because grassroot organizations gain members and influence as a direct function of their efficacy, they provide the clearest test researchers have to date of the value of different approaches to enhancing public cooperation.

## Transparency

*Action Editor:* Dolores Albarracin

*Editor:* Interim Editorial Board

## Notes

1. Despite the potential for accelerated publication to result in low-quality research, as of 2022, the volume of retractions of COVID-19 research has been no higher than average (Retraction Watch, 2022; Waltman et al., 2021).

2. See, for example, https://coronavirus.jhu.edu/map.html and https://www.researchgate.net/topic/COVID-19.

3. See Australian Institute of Family Studies (2020), Queensland Government (2023), Imperial College London (2023), and Breakthrough Action + Research for Social Science Behavior Change (2023).

4. For COVID-19 misinformation policies, see Facebook (n.d.)., Instagram (n.d.)., and YouTube (2023).

5. Note that through their very name—“mutual-aid groups”—these subcommunities leveraged the human tendency to engage in reciprocal cooperation, even if most people who were offering help expected to receive few tangible benefits in return.

6. For example, see Unite Against COVID-19 (2023).

7. See Queensland Government (2022).
